# NMR analysis reveals significant differences in the plasma metabolic profiles of Niemann Pick C1 patients, heterozygous carriers, and healthy controls

**DOI:** 10.1038/s41598-017-06264-2

**Published:** 2017-07-24

**Authors:** Fay Probert, Victor Ruiz-Rodado, Danielle te Vruchte, Elena-Raluca Nicoli, Tim D. W. Claridge, Christopher A. Wassif, Nicole Farhat, Forbes D. Porter, Frances M. Platt, Martin Grootveld

**Affiliations:** 1 Department of Pharmacology, De Montfort University, Leicester, UK; 2 Department of Pharmacology, University of Oxford, Oxford, UK; 3 Section of Molecular Dysmorphology, Eunice Kennedy Shriver National Institute of Child Health and Human Development, National Institute of Health, Department of Health and Human Services, Bethesda, MD USA; 4 Department of Chemistry, University of Oxford, Oxford, UK

## Abstract

Niemann-Pick type C1 (NPC1) disease is a rare autosomal recessive, neurodegenerative lysosomal storage disorder, which presents with a range of clinical phenotypes and hence diagnosis remains a challenge. In view of these difficulties, the search for a novel, NPC1-specific biomarker (or set of biomarkers) is a topic of much interest. Here we employed high-resolution ^1^H nuclear magnetic resonance spectroscopy coupled with advanced multivariate analysis techniques in order to explore and seek differences between blood plasma samples acquired from NPC1 (untreated and miglustat treated), heterozygote, and healthy control subjects. Using this approach, we were able to identify NPC1 disease with 91% accuracy confirming that there are significant differences in the NMR plasma metabolic profiles of NPC1 patients when compared to healthy controls. The discrimination between NPC1 (both miglustat treated and untreated) and healthy controls was dominated by lipoprotein triacylglycerol ^1^H NMR resonances and isoleucine. Heterozygote plasma samples displayed also increases in the intensities of selected lipoprotein triacylglycerol ^1^H NMR signals over those of healthy controls. The metabolites identified could represent useful biomarkers in the future and provide valuable insight in to the underlying pathology of NPC1 disease.

## Introduction

Niemann-Pick type C (NPC) disease is an autosomal recessive, neurodegenerative lysosomal storage disorder, which presents with a range of clinical phenotypes^1^. The hallmark of NPC disease is a complex storage pattern of multiple lipid species within lysosomes and late endosomes (LEs). Studies suggest that this process is triggered by the storage of sphingosine within lysosomes, leading to depletion of lysosomal calcium ion levels, and the subsequent accumulation of glycosphingolipids and unesterified cholesterol^2^. In view of the wide range of clinical phenotypes and the rarity of NPC1 disease (an estimated frequency of 1.12:100,000 live births)^3, 4^, diagnosis can represent a significant challenge. Historically, the most effective diagnostic test for NPC involved filipin staining of fibroblast cultures established from skin biopsies which, although accurate, is difficult to obtain, invasive, time-consuming, and often only available at specialised facilities. Filipin specifically binds to unesterified cholesterol, allowing the accumulation of cholesterol in perinuclear vesicular compartments to be evaluated. Unfortunately, the extent of cholesterol accumulation in fibroblasts does not correspond to clinical severity, and even the most severely affected patients may fail to be diagnosed by this approach. Indeed, this method gives inconclusive results in *ca*. 15% of cases^5^, and genomic sequencing of the *NPC1* and *NPC2* genes is recommended in order to confirm diagnosis. However, genotypic screening is insensitive and, in some instances, can be complicated by the large number of sequence variants with unknown significance which give rise to the NPC phenotype. Consequently, confirming NPC diagnosis via genotyping has some limitations. Indeed, routine exomic-based sequencing fails to identify mutations in 10% of patients presenting with the NPC phenotype (although additional disease-causing genes cannot be excluded)^6^.

In view of the difficulties associated with NPC diagnosis, the search for a novel, NPC-specific biomarker (or set of biomarkers) is a topic of much interest. Several potential serum biomarkers have been discovered to date, including glycoprotein nonmetastatic melanoma protein B^7^, the pro-inflammatory proteins galectin-3 (LGALS3) and lysosomal aspartic protease cathepsin D (CTSD)^8^, oxysterols (cholestane-3β,5α,6β-triol and 7-ketocholesterol)^6, 9–12^, monohexosylceramide, ceramide^13^, and lysosphingomyelin^14, 15^. In addition, 3β-sulfooxy-7β-N-acetylglucosaminyl-5-cholen-24-oic acid, along with its glycine and taurine amide conjugates, have been highlighted as potential biomarkers in urine^16^. To the best of our knowledge, to date no NMR-based investigations of NPC1 blood plasma have been conducted, although we have previously investigated the ^1^H NMR urinary profiles of NPC1 patients^17^. Despite the identification of a number of potential biomarkers of NPC1 disease in urine, to date no diagnostic test has been fully validated for translation to the clinic. While urine analysis is less invasive than blood analysis, blood samples are still easily obtained and with only minimal discomfort. Thus, a blood based diagnostic test which is still of clinical use would be less invasive than the current ‘gold standard’ skin biopsy test described above^5^. The high (and extremely variable) water content of urine, along with significant variations in pH in particular, can render this biofluid difficult to analyse by NMR-based metabolomics approaches. In contrast, plasma is far less affected by external factors such as diet, and homeostatic regulation ensures that inter-sample variation is minimal. We therefore elected to explore the metabolomics profiles of blood plasma samples collected from NPC1 disease patients not only to identify novel potential biomarkers, but also to provide further insights in to the underlying pathology of this debilitating condition.

The intrinsically quantitative and untargeted nature of high-resolution NMR analysis has the advantage of providing detailed information on a range of metabolites simultaneously with the need for only minimal sample preparation. The resulting metabolic profiles are extremely information-rich and, using associated multivariate (MV) exploratory data analysis and pattern recognition techniques, can discriminate between disease states without the requirement for direct identification of individual compounds. Such distinctive metabolic patterns, which are representative of the disease can serve as a more powerful diagnostic tool than the measurement of a single biomarker in isolation.

Therefore, in this study we have employed high-resolution proton (^1^H) NMR-linked metabolomics strategies to profile plasma samples collected from an extensive cohort of patients with NPC1 disease. Random Forests (RFs) analysis was employed to compare the plasma profiles of NPC1 patients with those of both healthy controls and heterozygous carriers. In addition, the influence of miglustat treatment on the ^1^H NMR plasma profiles of NPC1 patients was investigated. The metabolic and potential clinical significance of the results acquired are discussed in detail.

## Results

### Overview

In total, 225 plasma samples were included in this study, details of which are summarised in Table 1. Overall, 75 plasma samples were collected from 40 untreated NPC1 patients (NPC1), and 89 samples from 34 NPC1 patients undergoing treatment with miglustat (MGS). Typically, samples were collected from each patient twice over the course of 2 years, with an average of 7 months between resampling. Moreover, 31 samples collected from heterozygous parents and grandparents (HET classification), along with 30 healthy control samples (HC) were analysed.

**Table 1:** Clinical characteristics of NPC1 patients and healthy control subjects included in this cohort.

	NPC1 untreated	NPC1 MGS treated	Heterozygous parents	Healthy controls
Number (male/female/unknown)	75 (42/33)	89 (54/35)	31 (15/13/3)	30 (23/6/2)
Average age in years (range)	19 (0.3–54)	11 (1–28)	N/A	15 (0.4–50)
ASIS range	0.13–5.97	0.07–9.16	N/A	N/A

**Figure 1:**
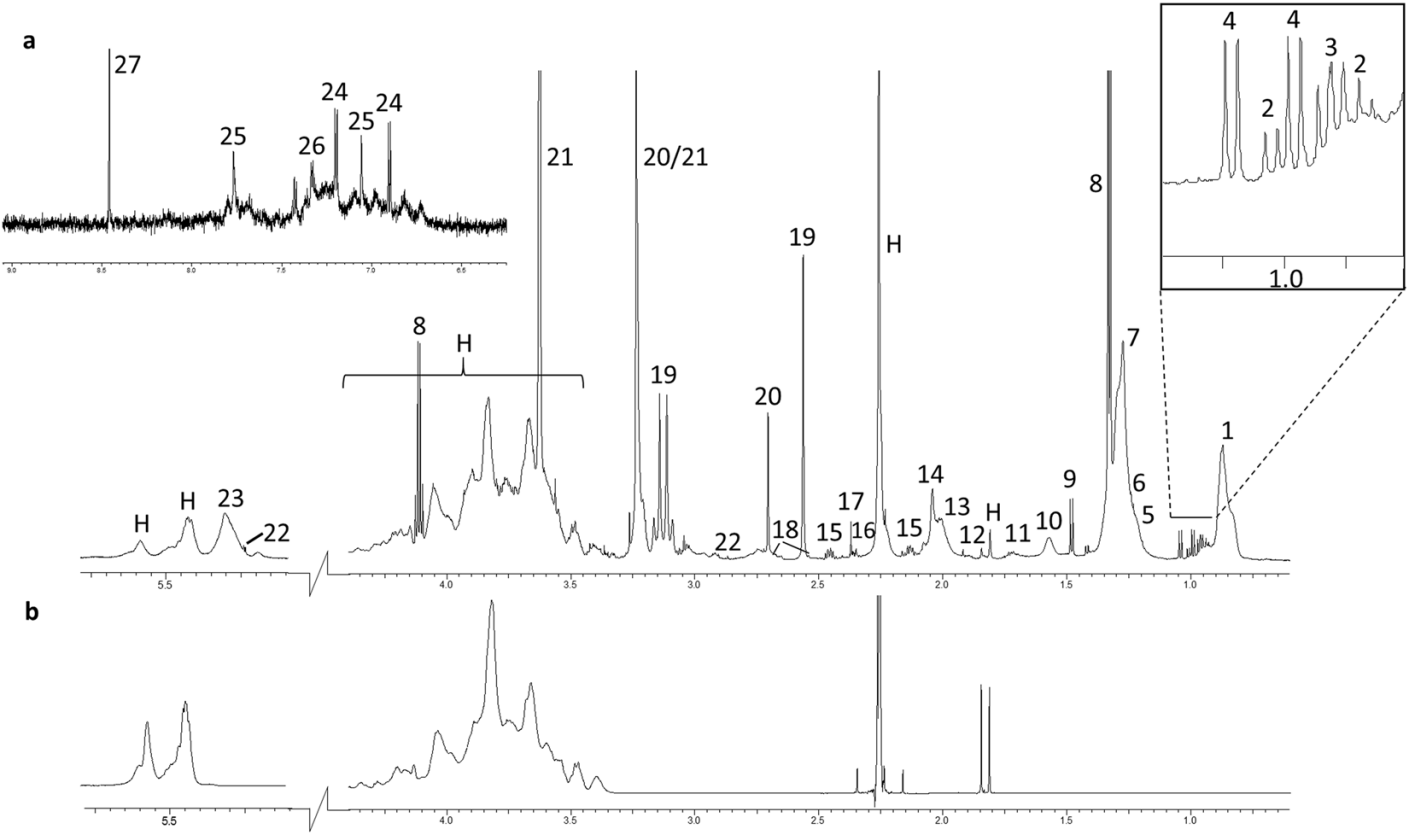
Representative ^1^H CPMG NMR spectrum of (**a**) untreated NPC1 patient plasma with 27 major metabolites labelled; (1) mobile lipid -CH_3_, (2) isoleucine, (3) leucine, (4) valine, (5) 2,3 butanediol, (6) 3-hydroxybutyrate, (47) mobile lipid -(CH_2_)_n_-, (8) lactate, (9) alanine, (10) mobile lipid –C**H**_**2**_CH_2_CO, (11) arginine, (12) acetate, (13) proline/mobile lipid CH_2_-C**H**_**2**_-CH=, (14) N-acetyl glycoprotein/mobile lipid CH_2_-C**H**_**2**_-CH=, (15) glutamate, (16) glutamine, (17) acetoacetate, (18) citrate,(19) Ca^2+^-EDTA, (20) Mg^2+^-EDTA, (21) EDTA, (22) Zn^2+^-EDTA, (22) α-glucose, (23) mobile unsaturated lipids >CH=CH<, (24) tyrosine, (25) histidine, (26) phenylalanine, (27) formate, and (H) contaminants arising from histopaque cell separation. (**b**) ^1^H NMR spectrum of histopaque column flow through; matching contaminants are clearly visible in the plasma spectrum.

A typical, representative ^1^H-NMR CPMG spectrum of plasma collected from an untreated NPC1 patient is shown in Fig. 1a. Several artefactual broad resonances were observed in all samples within the 1.80–1.86, 2.18–2.31, 3.3–4.5, and 5.37–5.68 ppm regions, and these were attributed to contaminants arising from the histopaque separation procedure. These signals included those arising from sodium diatrizoate and polysucrose. Analysis of a ^1^H NMR spectrum of a flow-through sample collected from the histopaque column confirmed this (Fig. 1b). Unfortunately, the level of contamination in each sample varied significantly, and therefore subtraction of the histopaque flow-through spectra from those of plasma did not allow quantification of the underlying resonances (Figure S1). Consequently, all regions of the spectra containing resonances attributable to histopaque contaminants were eliminated from further analysis.

In order to understand the inter-sample variation (which encompasses both the biological and technical variation) for each disease classification explored (NPC1, HET, MGS, and HC), the spectral relative standard deviation was computed as previously described^18^ (Figure S2a). The median RSD values are 25.2%, 23.7%, 22.2%, and 29.2% for the NPC1, MGS, HET and HC groups respectively, and hence are consistent with previous reports^19^. These data confirmed that there were no significant differences between the spectral RSDs of each disease classification, an observation suggesting that disease or treatment status does not influence inter-individual variability.

There was imperfect gender- and age matching between the healthy control and NPC1 patient samples (Figure S2b and Table 1). However, no significant differences were observed between the ^1^H NMR spectral profiles of male and female plasma by univariate analysis (t-test with correction for multiple comparisons), and no separations were observed by principal component analysis (PCA) (Figure S3a and b). Similarly, variation arising from the age covariable was investigated by splitting the cohort in to two groups: firstly ‘young’, operationally defined as less than 10 years of age, and secondly ‘older’, i.e. participants greater than or equal to 10 years of age. Again, no significant differences were observed between these two groups (Figure S3c), and no separation in the PCA scores plots was evident (Figure S3d). A small decrease in the -CH_3_ (very low density lipoprotein [VLDL]) region of the spectra is observed in the ‘young’ cohort when compared to the ‘older’ one; however, this difference did not attain statistical significance (Figure S3c). In addition, MV analysis of covariance (MANCOVA) using disease classification, gender, age, and a gender-age first-order interaction term confirmed a significant disease class effect on the ^1^H NMR variables (*p* value < 0.01), but no significant ones ascribable to gender or age.

### Multivariate analysis is able to distinguish between the plasma NMR metabolic profiles of untreated NPC1 patients and healthy controls

**Figure 2:**
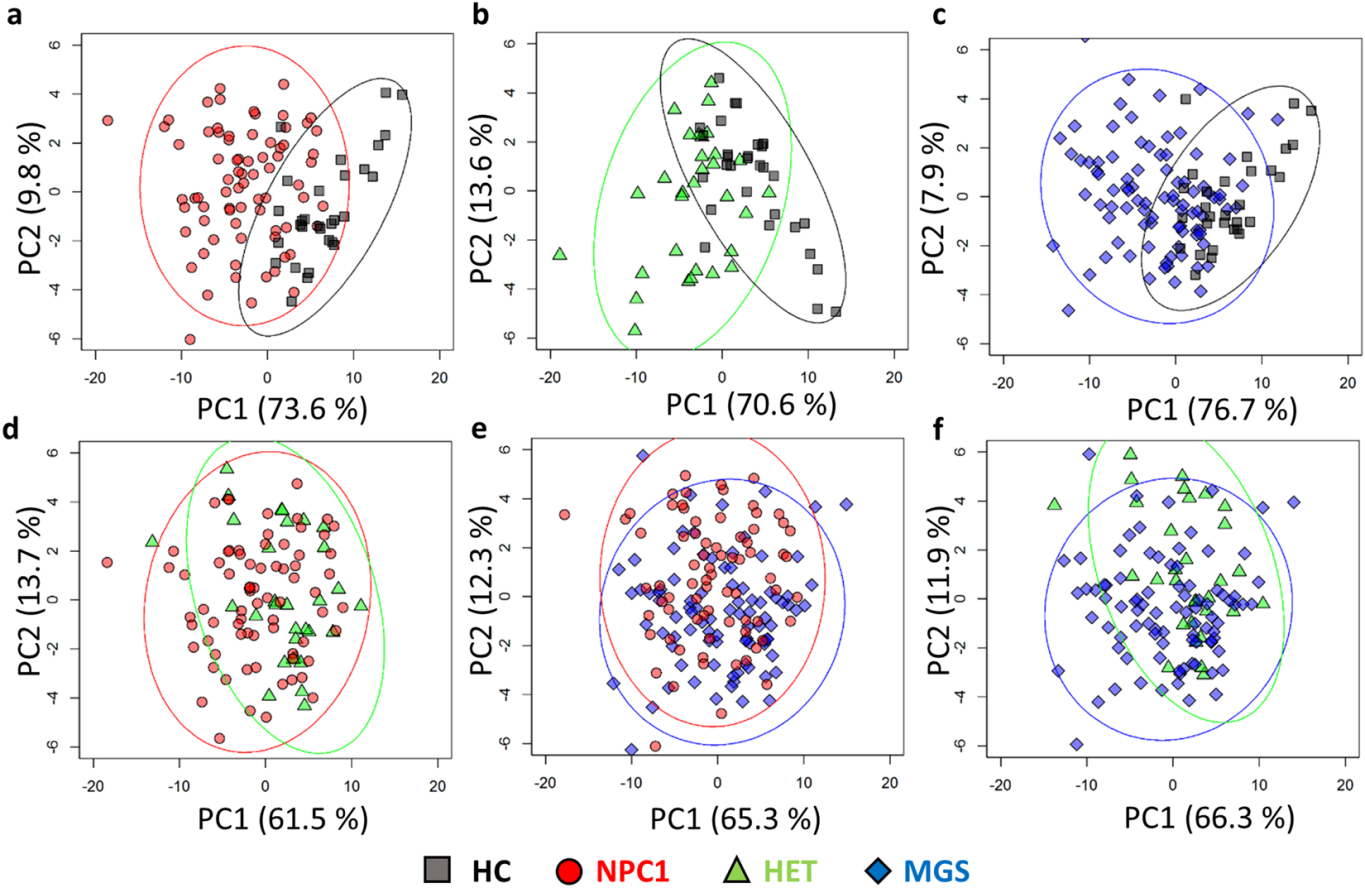
Principal component analysis scores plots (component 1 *vs*. component 2) of comparisons made on (**a**) HC *vs*. NPC1, (**b**) HC *vs*. HET, (**c**) HC *vs*. MGS, (**d**) NPC1 *vs*. HET, (**e**) NPC1 *vs*. MGS, and (**f**) and MGS *vs*. HET.

Inspection of the PCA scores plots revealed two distinct clusters for the untreated NPC1 patients (NPC1) and healthy controls (HC), indicating that the ^1^H NMR plasma metabolic profiles of untreated NPC1 patients are clearly distinct from those of healthy individuals (Fig. 2a). Corresponding loadings for PC1 and PC2 are available in the supplementary material (Figure S4). The mobile lipoprotein -(CH_2_-)_n_ VLDL, -CH_3_ VLDL, and lactate spectral regions load strongly on component one suggesting a correlation. In addition, -(CH_2_-)_n_ VLDL loads strongly in the positive direction of component 2 while lactate, Mg^2+^-EDTA, and Ca^2+^-EDTA load strongly in the negative direction suggesting an inverse correlation of these metabolite resonances. In order to determine which metabolites are responsible for this discrimination, and also develop a predictive model with diagnostic potential, a RFs model was constructed, and this gave an OOB (Out-Of-Bag) error of 0.089 ± 0.002 (mean ± SEM). Therefore, the NPC1 *vs*. HC RFs analysis indicated that this model is able to classify NPC1 and HC ^1^H NMR plasma profiles with high levels of accuracy, sensitivity, and specificity (Table 2).

**Table 2:** Random forest classification performances of training and test sets. Mean (EEM).

	Out-of-bag error	Accuracy	Sensitivity	Specificity
HC v. NPC1	0.089 (0.002)	0.910 (0.004)	0.923 (0.005)	0.887 (0.012)
HC v. HET	0.175 (0.005)	0.818 (0.007)	0.824 (0.011)	0.823 (0.012)
HC v. MGS	0.160 (0.003)	0.832 (0.006)	0.864 (0.007)	0.729 (0.015)
NPC1 v. HET	0.205 (0.003)	0.789 (0.005)	0.808 (0.007)	0.747 (0.018)
NPC1 v. MGS	0.353 (0.004)	0.646 (0.006)	0.664 (0.008)	0.632 (0.010)

**Figure 3:**
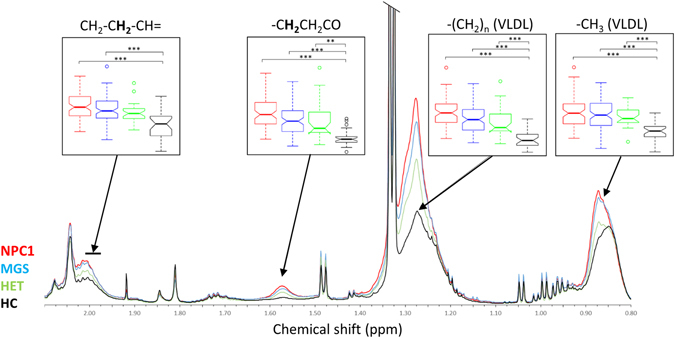
Lipid region (0.8–2.1 ppm) of average ^1^H NMR spectra of NPC1 (red), MGS (blue), HET (green), and HC (black) plasma. Box plots illustrate the full range, interquartile range and median of the spectral integral of each region given (Bonferroni-corrected p-values < 0.05, 0.01, 0.001 are represented by *, ** and *** respectively).

The most important (top 8) discriminatory variables (intelligently-selected -^1^H NMR buckets) selected by the RFs analysis, along with their corresponding assignments are listed in Table 3. The discriminatory variables are ranked in order of importance based on the mean decrease in accuracy (MDA) value. The MDA is calculated by removing each variable from the RF model and calculating how much the accuracy of the model decreases. The variable which gives rise to the largest decrease in accuracy when removed is ranked as the most important variable, and so on. Inspection of the MDA values indicates the number of variables required for an effective discrimination between groups; variables causing little or no difference to the MDA value were excluded (in this case only eight variables were required for discrimination). As expected (because defects in lipid homeostasis are a key clinical manifestation of NPC1)^6^, differences in lipoprotein-associated lipid resonances were important in discriminating between untreated NPC1 patients and healthy controls. Most notably, NPC1 patients exhibited an increase in the lipid bulk chain (-CH_2_-)_n_ of VLDL and low density lipoprotein (LDL), terminal -CH_3_ of VLDL, -C**H**_**2**_CH_2_CO, CH_2_-C**H**_**2**_-CH=, and unsaturated >CH=CH< triacylglycerol NMR spectral region integrals when compared to HC. In addition, isoleucine concentration was significantly increased in NPC1 patient plasma samples. All of the important discriminatory variables identified by the RFs approach were also identified as significantly increased in a univariate context (*p* < 0.001 following correction for multiple comparisons, Table 3), and a full list of all significantly modified variables, together with fold-changes, is available in Table S1. Indeed, Fig. 3 shows that there is a clear increase in the mean intensities of the above lipid resonances in NPC1 patients over those of healthy controls.

**Table 3:** Significant random forest discriminatory variables.

Metabolite	Bin (ppm)	NPC1 *vs*. HC	HET *vs*. HC	MGS *vs*. HC	NPC1 *vs*. HET
**Lipids**					
-CH_3_ (HDL)	[0.81 .. 0.83]				**↓(3)**
-CH_3_ (VLDL)	[0.83 .. 0.89]	↑***(7)	↑***(2)	↑***(6)	
-(CH_2_-)_n_ (HDL)	[1.21 .. 1.23]		↑***(7)		**↓ (9)**
-(CH_2_-)_n_ (LDL)	[1.23 .. 1.25]	↑***(8)	↑***(3)	↑***(4)	
-(CH_2_-)_n_ (VLDL)	[1.25 .. 1.31]	↑***(2)	↑***(5)	↑***(1)	**↑(8)**
-C**H**_**2**_CH_2_CO	[1.53 .. 1.61]	↑***(1)	↑**(8)	↑***(3)	**↑(2)**
CH_2_-C**H**_**2**_-CH= (exclusively unsaturated lipid function)	[1.96 .. 2.05]	↑***(5)			
Unsaturated lipid >CH=CH< (bin 1)	[5.26 .. 5.32]	↑***(3)	↑***(1)	↑***(2)	
Unsaturated lipid >CH=CH< (bin 2)	[5.32 .. 5.37]	↑***(4)	↑***(4)	↑***(5)	**↑ (6)**
**Amino Acids**					
Isoleucine	[0.913 .. 0.936]	↑***(6)			**↑(4)**
Proline/CH_2_-C**H**_**2**_-CH= (bin 1)	[1.94 .. 1.965]		↑***(9)		
Proline/CH_2_-C**H**_**2**_-CH= (bin 2)	[1.95 .. 1.96]		↑***(6)		
Histidine (bin 1)	[7.02 .. 7.08]				**↑(7)**
Histidine (bin 2)	[7.74 .. 7.85]				**↑ (5)**
**Metal ions**					
Ca^2+^-EDTA	[2.52 .. 2.58]			↑***(7)	↑*(1)
**Other**					
2,3-butanediol	[1.13 .. 1.15]			**↑(8)**	

Arrows indicate an increase/decrease in the measured metabolite with respect to the HC samples or with respect to the HET samples in the case of the NPC1 v. HET comparison. The results of the Tukey’s HSD test for each metabolite identified by the random forests analysis are indicated by an asterisks (Bonferroni corrected *p*-values < 0.05, 0.01, 0.001 are represented by *, **, and *** respectively). The order in which the discriminatory variables were ranked by random forest analysis based on their MDA value is represented in brackets (1 = discriminatory variable with highest MDA).

Whilst it is well known that changes in lipid homeostasis occur in NPC1 disease, the extent of these changes do not appear to correlate with disease severity, and clearly are not unique to NPC1 disease^20, 21^. Therefore, in an attempt to identify metabolites which may have diagnostic value specific to NPC1 disease, and also offer insights into perturbed metabolic pathways therein, the RFs analysis was repeated with all lipid resonance bucket regions excluded. In the absence of contributions from these resonances, an OOB error value of 0.216 ± 0.004 was achieved, with accuracy, specificity and sensitivity values of 0.784 ± 0.007, 0.814 ± 0.007, and 0.716 ± 0.018 respectively. While this model is not as robust, it nevertheless offers a reasonable classification performance. The metabolites responsible for discrimination of NPC1 patients and controls in the absence of lipid resonances were (in descending order of MDA) isoleucine and alanine, both of which were found to be significantly increased in the NPC1 classification by univariate analysis (*p* < 0.001 Table S1). However, in view of the nature of the CPMG NMR sequence used, contributions from broad lipid signals cannot, of course, be discounted in the isoleucine region of the spectra. Measurement of additional isoleucine spectral regions with no lipoprotein overlap was prevented by the histopaque contaminant present in these samples.

### Multivariate techniques are unable to distinguish between MGS-treated and untreated NPC1 patient plasma NMR metabolic profiles

Since MGS is currently the only EMA approved therapy for the treatment of NPC1 disease, we therefore investigated if treatment with MGS gave rise to changes to the ^1^H NMR plasma metabolic profile of NPC1 patients. No distinct clustering of the dataset ascribable to the NPC1 and MGS classifications were observed in PCA scores plots obtained (Fig. 2e), and a RFs analysis (Table 2) was unable to discriminate between MGS-treated (MGS) and untreated (NPC1) patient plasma samples. However, a clear separation was observed between MGS-treated NPC1 and the healthy control (HC) samples (Fig. 2c), and many of the same discriminatory variables responsible for the distinction of untreated NPC1 and healthy control groups were also identified in this analysis (Table 3). In particular, ^1^H NMR spectra from the MGS-treated group also showed significant increases (by univariate analysis) in lipoprotein-associated triacylglycerol resonances when compared to HC controls (Fig. 3). This suggests that the ^1^H NMR plasma metabolic profiles of MGS-treated NPC1 patients is similar to that of untreated NPC1 patients. However, the isoleucine-CH_3_ function bucket was not selected as a discriminatory variable for MGS-treated samples in this comparison. Moreover, out of all of the metabolites present in the NMR spectra acquired, some additional variables were required for discrimination between MGS-treated patients and healthy controls, i.e. those that were not selected by the RFs model which compared untreated NPC1 patients with the controls. These included increases in the Ca^2+^-EDTA and 2,3-butanediol resonances.

### The plasma NMR metabolic profiles of the heterozygous (parental) classification are distinct from those of healthy controls

Prominent clustering in the PCA scores plot incorporating only the HET and HC groups was observed (Fig. 2b). This indicates that, whilst heterozygous carriers do not exhibit the pathological features of NPC1 disease, the plasma NMR metabolic profiles remain distinct from those of HC. Indeed, RFs analysis was able to successfully discriminate between the ^1^H NMR spectra of plasma collected from these two classifications with an accuracy of 82% (Table 2). Similar to the NPC1 patients, the key variables responsible for discrimination between HET and HC samples are dominated by lipoprotein-associated triacylglycerol resonances (Fig. 3 and Table 3). The CH_2_-C**H**_**2**_-CH= unsaturated lipid variable, which was selected as adiscriminatory one between the NPC1 and control spectra, was not identified in the HET *vs*. HC analysis, although an increase in the overlapping proline/CH_2_-C**H**_**2**_-CH= spectral region was observed only for the HET samples.

### The plasma NMR metabolic profiles of the heterozygous, parental controls are distinct from those of NPC1 patients

There was, however, some evidence of separation between the plasma ^1^H NMR metabolic profiles of the heterozygous parents and those of NPC1 patients (Figs 2 and 3). Indeed, the RFs analysis performed was able to discriminate between these two classes (Table 2), but with an accuracy of only 79%. Interestingly, while the discriminatory variables responsible for separating NPC1 from HC samples predominantly arise from lipoprotein triacylglycerol signals, the most important variable for discrimination of NPC1 from HET samples is an increase (in NPC1 relative to HET) in the Ca^2+^-EDTA signal (indicating an elevated Ca^2+^ ion level), followed by a decrease in that of the high-density-lipoprotein (HDL)-triacylglycerol terminal-CH_3_ resonance.

### Investigation of inter-relationships between plasma lipoprotein triacylglycerol resonance intensities and triacylglycerol-normalised total and lipoprotein-associated cholesterol concentrations

PCA analysis of only the lipoprotein triacylglycerol ^1^H NMR resonances normalised to that of the total triacylglycerol-C**H**_**3**_ function signal revealed that the variables were clearly segregated into four major orthogonal components (loading scores vectors in brackets): (1) PC1, encompassing the VLDL-(C**H**_**2**_)_n_- function (0.91), the triacylglycerol-C**H**_**2**_CO- group (0.88), and both vinylic proton triacylglycerol signals 1 and 2 (0.83 and 0.92 respectively); (2) PC2, involving HDL-C**H**_**3**_ and -(C**H**_**2**_)_n_- signals (0.95 and 0.91 respectively), and the VLDL-C**H**_**3**_ function (−0.93); (3) PC3, exclusively featuring the triacylglycerol-CH_2_-C**H**_**2**_-CH= resonance alone (0.96); and (4) PC4, involving the LDL-associated triacylglycerol-(C**H**_**2**_ **)**_**n**_ **-** signal alone (0.97). Hence, this strategy appeared to successfully segregate resonances arising from different lipoproteins (although it should be noted that there is at least some overlap between these lipoprotein triacylglycerol signals for each functional group where they are partially distinguishable), i.e., both HDL resonances are strongly loaded on PC2, and the single partially NMR-distinguishable LDL-(-C**H**_**2**_-)_n_ signal loaded exclusively on PC4.

A corresponding PCA analysis then performed on the clinical chemistry lipid profile dataset (all variables this time normalised to total triacylglycerol concentrations prior to analysis) demonstrated that the three variables investigated were predominantly segregated into two orthogonal components, the first (PC1*) containing the total cholesterol and LDL-cholesterol (LDL-c) variables (loading scores vectors 0.80 and 0.93 respectively), the second (PC2*) HDL-cholesterol (HDL-c) alone (0.91), as might be expected in view of the commonly observed higher LDL- to HDL-cholesterol concentration ratios of blood plasma samples. Clearly, all these variables very strongly loaded on the each of the PCs featured.

Finally, we performed canonical correlation analysis (CCorA) of the plasma sample scores vectors arising from the above two sets of lipidomic PCA analyses, i.e. PC1-PC4 for the ^1^H NMR dataset, and PC1* and PC2* for the clinical chemistry one. This approach avoids multicollinearity problems since the PC scores vectors analysed are orthogonal (i.e., uncorrelated).

**Figure 4:**
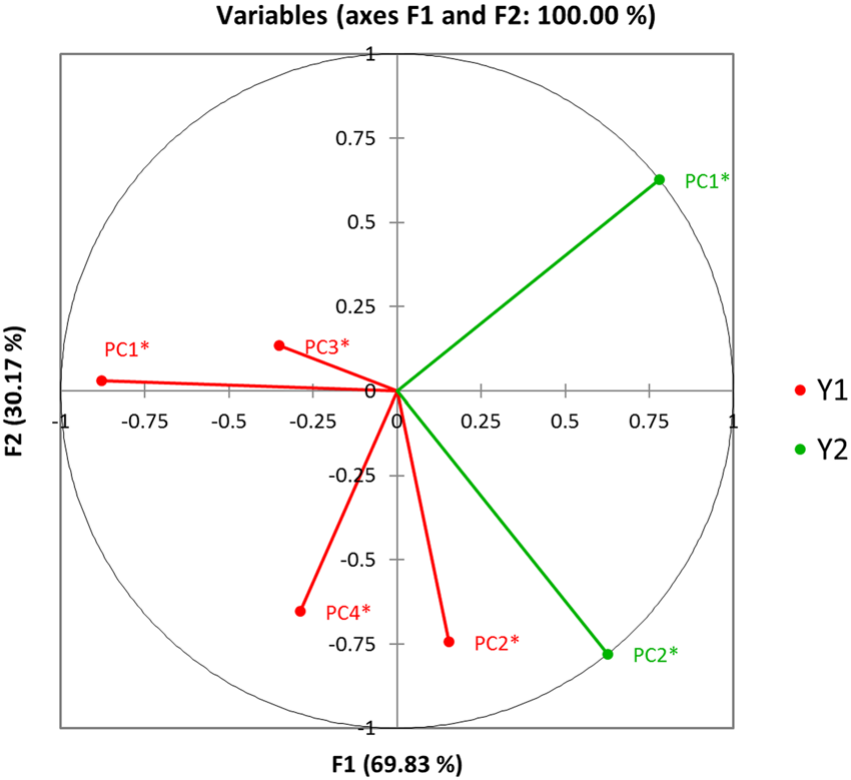
Canonical correlation analysis (CCorA) plot revealing associations between the PC scores vectors arising from (1) total lipoprotein triacylglycerol-CH_3_ function-normalised ^1^H NMR triacylglycerol resonances (PCs 1–4), and (2) total triacylglycerol concentration-normalised clinical chemistry-determined total, LDL- and HDL-associated cholesterol concentrations (PCs 1* and 2*). Information regarding the predictor variables loading on each set of PCs is provided in section 2.6. Y1 and Y2 represent scores vector datasets arising from the separate ^1^H NMR and clinical chemistry datasets respectively.

This analysis revealed that (1) the PC2* scores vectors positively correlated with those of PC2, as might be expected since they both have HDL sources and communalities, and (2) PC1* was anti-correlated with PC4, i.e. a linear combination of plasma triacylglycerol-normalised total cholesterol and LDL-cholesterol concentrations was negatively correlated with the ^1^H NMR PC arising from LDL-triacylglycerols alone, and this indicates a negative correlation between plasma LDL-cholesterol and LDL-triacylglycerol levels. Figure 4 displays a correlation plot of these inter-relationships.

## Discussion

Data presented here represent the first detailed comparative ^1^H NMR-based metabolomics investigation of plasma collected from NPC1 patients (both untreated and MGS-treated), their heterozygous parental carriers, and healthy controls. Since the samples utilised in this study were first used for a LysoTracker-based investigation^22^, blood samples were collected into EDTA-containing collection tubes, and the plasma arising therefrom separated using a histopaque column (to allow simultaneous cell separation). Plasma samples were then stored at −80 °C and retrospectively analysed by ^1^H NMR spectroscopy at an operating frequency of 700 MHz. This resulted in histopaque contaminants in the NMR plasma samples, which were confirmed via the acquisition of a ^1^H NMR spectrum of a histopaque flow-through sample (Fig. 1b). Subtraction of the histopaque flow-through resonances from the plasma spectra, however, did not reveal any additional resonances masked by this contamination (Figure S1). As a consequence, these regions of the spectra were omitted from our analysis, a process unfortunately resulting in a significant loss of information from a large number of metabolite resonances, including those ascribable to the molecularly-mobile-N^+^(CH_3_)_3_ function of HDL, free choline, betaine, acetoacetate, and glucose. In addition, strong signals arising from the EDTA anticoagulant obscured most of the citrate resonances in the spectra, but provided information on the metal ion levels in plasma, which allowed Ca^2+^-EDTA and Mg^2+^-EDTA complex resonances to be included in the analysis. While these resonances provide an indirect measure of the metal ion composition of the plasma samples studied, it should be noted that analysis of these metal ion-EDTA chelate resonances (the ethylenic group protons in this case) represents a composite of those complexed by plasma proteins and organic acid anions such as citrate^23^. Although a significant region of the ^1^H NMR spectrum was excluded in view of the histopaque contaminant, a large amount of information could still be extracted from the NMR datasets acquired. Indeed, NPC1 patients were successfully differentiated from healthy controls based on their plasma NMR metabolic profiles with an accuracy of 91%. Investigation of the variables responsible for this discriminatory model allows identification of the specific metabolites perturbed in NPC1 disease plasma which may, with further investigation, provide information on the underlying pathological processes and may be useful biomarkers.

A central feature of NPC1 disease is impaired lipid intracellular-transport, leading to an accumulation of glycosphingolipids and cholesterol in lysosomes and late endosomes, and therefore modifications to the plasma concentrations of lipoproteins and their molecularly-mobile, spin-echo Fourier transform NMR-detectable triacylglycerols may be expected. Indeed, it has been shown that a decrease in HDL-c is the most common lipoprotein abnormality amongst NPC1 patients, together with decreased LDL-c and increased triacylglycerol (TG) concentrations^24, 25^. Since the -(CH_2_)_n_, -C**H**_**2**_CH_2_CO, and N-acetylglycoprotein-NHCOC**H**_**3**_/CH_2_-C**H**_**2**_-CH= mobile lipid regions of the ^1^H NMR spectral profiles have been previously shown to be correlated with plasma TG levels in both humans and mice^26, 27^, this is consistent with the increased lipoprotein-associated TG spectral regions observed in this study. The HET classification samples also displayed significant increases in plasma lipid resonances, and the RFs analysis strategy was able to discriminate between the HET and HC groups with a high level of accuracy (82%, Table 2). This was somewhat surprising, since *NPC1* heterozygous carriers do not display any disease pathology, and hence we might expect the plasma ^1^H NMR profiles of these sampling groups to be indistinguishable from those of age-matched healthy controls. Interestingly, the heterozygous carrier classification samples exhibit similar increases in the triacylglycerol terminal–CH_3_, bulk chain (-CH_2_-)_n_, and vinylic proton spectral buckets as the NPC1 samples, with the exception of CH_2_-C**H**_**2**_-CH= region which was not selected as a discriminatory variable for distinguishing the HET spectra (Table 3). This suggests that the heterozygote carrier group may also exhibit increased plasma TG levels, an observation also indicating that increased TG levels are not directly linked to disease pathology (which is consistent with the lack of correlation between TG and disease severity/age of death noted in previous reports)^24^.

It is also clear from the univariate and RFs analysis performed that the plasma NMR metabolic profiles of heterozygotes are distinct from those of healthy controls. Our RFs model was able to discriminate between the metabolic profiles of the HET and NPC1 samples, although the error rate is larger (OOB = 0.21, Table 2). This suggests that while the metabolic profiles of NPC1 and HET samples are similar (dominated by increases in lipid resonances), there are some further, relatively minor differences which the MV analysis is also able to detect - these differences may provide information on metabolites correlated with the pathology of NPC1 disease. While the terminal-CH_3_ and bulk chain–(CH_2_-)_n_ HDL regions of the spectra do not serve as significant discriminatory variables in the NPC1 *vs*. HC analysis, they are important for distinguishing between NPC1 patients and the heterozygous carriers, with a decrease in intensity of these buckets observed in the NPC1 cohort (Table 3). Decreases in HDL-c concentrations have been shown to correlate with age at death in a small number of NPC1 patients, and has also been postulated to represent a useful biomarker for the monitoring of treatment response^24^. However, blood plasma HDL-c levels are not unique to NPC1 disease, and have been observed in other lysosomal storage disorders such as cholesterol ester storage disease^21^, together with many further non-lysosomal diseases^28–30^.

Although the RFs analysis was unable to distinguish between untreated and MGS-treated NPC1 patients (accuracy of only 65%, Table 2), box plots of the spectral intensities of the lipid regions, along with an investigation of the average spectra for each disease class (Fig. 3), revealed a trend, with the MGS-treated samples exhibiting lower lipid levels than those of the untreated NPC1 patients, and with the heterozygous carrier samples lower still. This is consistent with a previous report that miglustat treatment reduces TG plasma levels in patients with Gaucher’s disease^31^.

FDR-corrected univariate analysis revealed that the MGS-treated NPC1 disease group had significantly elevated lipoprotein-associated triacylglycerol resonance intensities over those of the HC control group (Table S1). However, not all these differences attained statistical significance when the dataset was analysed according to our RF multivariate model system (Table 3). In view of these observations, it should be noted that univariate methods simply consider the mean and variance values of single variables, whereas multivariate analysis strategies encompass all variables simultaneously, and therefore focus on simultaneous relationships (linear or otherwise), amongst all such variables available. Indeed, such multivariate approaches focus on covariances/correlations between these variables, and therefore they capture the extent of these relationships between potentially large numbers of variables. Therefore, for the variables concerned [HDL triacylglycerol (-CH_2_-)_n_, and total unsaturated -CH_2_-CH_2_-CH= functions], this observation may be ascribable to the ‘masking’ of information by relatively large numbers of uninformative variables, a lack of overlap between the univariate and MV testing approaches utilised, and/or complications with the provision of reliable estimates of covariances, notably when there are large numbers of potential predictor variables and relatively smaller sample sizes (although in this case there was a very favourable high sample size to NMR chemical shift bin variable ratio, i.e. *ca*. 6:1, 225 plasma samples and only 38 bin variables, unlike those of the great majority of other NMR-based metabolomics investigations currently performed).

Whereas the HDL lipid regions of the spectra were identified as discriminatory variables in the NPC1 *vs*. HET RFs model, the most important discriminatory variable was plasma Ca^2+^ concentration, which was monitored as its EDTA complex. Indeed, the Ca^2+^-EDTA resonance is significantly elevated (*p*-value < 0.05 following correction for multiple comparisons) in NPC1 patient plasma when expressed relative to that of the HET group (Table 3 and Figure S5). Whilst plasma Ca^2+^ level is not selected as a discriminatory variable for the NPC1 *vs*. HC comparison, the MGS-treated samples have an increased mean Ca^2+^-EDTA complex resonance intensity when compared to that of untreated NPC1 patients (Figure S5). Taken together, this suggests that investigation of plasma Ca^2+^ levels in NPC1 patients may be valuable. However, future work to verify this finding via other methods is required.

Although the variables selected by the RFs analysis for discriminating between the untreated and treated NPC1 groups are dominated by lipoprotein-associated TG resonances, significant differences in many amino acid spectral buckets were also observed (Tukey’s *hsd* test with correction for multiple comparisons, Table S1). The isoleucine, alanine, arginine, proline/CH_2_-C**H**_**2**_-CH=, and glutamine regions of both NPC1 and MGS spectra are increased relative to those of the HC samples (all Bonferroni-corrected *p*-values < 0.001). These modulations to plasma amino acid levels observed may partially reflect liver disease^32^, and hence may be ascribable to some level of hepatic dysfunction in NPC1 patients. Acute liver dysfunction can occur during the neonatal period, but low level chronic dysfunction may also occur in patients beyond this stage of development^33^, although the frequency of this is relatively low. The catabolic pathways of branched chain amino acids (BCAAs) are analogous to the fatty acid oxidation pathway, and can be utilised for lipid synthesis, since all three BCAA pathways ultimately generate propionyl-CoA and/or acetyl-CoA^34^. The intensity of the BCAA spectral buckets (containing overlapping resonances from leucine, isoleucine, and valine) were found to be significantly increased in spectra acquired on NPC1 patients over those of healthy controls. A potential modification to the BCAA degradation pathway in NPC1 disease has been previously reported^35^, and urinary concentrations of these metabolites were found to be elevated in untreated NPC1 patients when expressed relative to those of heterozygous carriers^17^.

Heterozygous carrier plasma does not display the same pattern of amino acid disruption as the NPC1 patients, with the only resonances significantly perturbed arising from the overlapping proline/CH_2_-C**H**_**2**_-CH= resonance (*p* < 0.001), an observation suggesting that modulation of the amino acid profiles observed in the plasma ^1^H NMR profiles of NPC1 patients may be linked to liver pathology associated with this disease. Indeed, a recent investigation conducted by our group revealed higher amino acids levels in hepatic tissue collected from a NPC1 mouse model. These increased levels of several amino acids likely arose from the liver parenchyma necrosis associated with hepatic fibrosis, a common feature in NPC1 liver in the mouse model^36^. However, contributions from lipoproteins in this region of the spectra cannot be discounted, and hence future work to investigate these potential amino acid imbalances in more detail is required.

It remains to be determined if these metabolic differences are specific to NPC1 or limited to detection of lysosomal/cholesterol storage diseases in general. Future work could involve the acquisition of high-resolution ^1^H NMR spectra of plasma from patients with other lysosomal storage diseases such as type 1 Gaucher and cholesterol ester storage (Wolman) diseases. Indeed, some of the major discriminatory features observed here included decreased HDL and increased VLDL concentrations, which are, of course, unspecific markers for NPC1 disease.

The samples analysed here were initially processed for a LysoTracker study, and retrospectively analysed by high-resolution ^1^H NMR spectroscopy (in view of the rarity of NPC1 disease and thus small numbers of available samples), and so a great deal of spectral information was lost in view of histopaque contamination. Future work following on from this study should make use of samples dedicated for ^1^H NMR analysis, collected into lithium-heparin tubes (in order to avoid the relatively large, interfering EDTA resonances) and omitting the histopaque separation step. In addition, there is imperfect gender and age matching between classes as no age-matched HET samples were available for this NPC1 cohort. Despite this, no significant differences were observed, and no models could be produced using these as factors. Finally, the NPC1 (both untreated and MGS-treated) and HET participants were fasted prior to plasma collection, whilst the HC control volunteers were not. One would expect lower lipid resonance intensities in plasma from fasted individuals. However, the converse was observed in the case of this cohort with the fasted NPC1 and HET spectra exhibiting significantly higher lipid resonance intensities than those of the controls. This suggests that the differences observed here in lipid resonance intensities may be even more apparent when a cohort of fasted controls is used. Despite these limitations, a great deal of information can be extracted from the ^1^H NMR profiles, and a predictive model with the ability to distinguish between NPC1 patients and healthy controls with high degrees of accuracy (91%) and specificity (85%) was generated. Data presented here therefore provide valuable information on the plasma metabolic profiles of NPC1 disease. Further investigation and validation of the discriminatory metabolites identified in this study may reveal novel biomarkers and provide valuable insight in to the underlying pathology of NPC1 disease.

## Conclusions

This preliminary investigation confirms that significant differences exist between the ^1^H NMR spectra of plasma collected from patients with NPC1 disease (both untreated and MGS-treated) and those of healthy control subjects, which are dominated by changes in their lipid profiles. In addition, the ^1^H NMR metabolic profile of plasma from corresponding heterozygous carriers was found to be distinct from those of both healthy controls and NPC1 patients, and this provides novel insights into the underlying mechanisms of this rare lysosomal storage disease.

## Materials and Methods

### Plasma sample collection

NPC1 patients included in this study were enrolled between 2006 and 2013 in an Institutional Review Board-approved, longitudinal, Natural History/Observational trial at the National Institutes of Health (06-CH-0186, NCT00344331) in accordance with all institutional Review Board guidelines and regulations. Written, informed consent was obtained for all subjects, and assent was obtained when possible. Clinical diagnosis was confirmed by filipin staining of fibroblasts and *NPC1* mutation analysis.

### Plasma and cell separation

Control samples were obtained by voluntary donation with informed consent, or from a commercial provider where informed consent/assent was given at the time of collection. The investigators were blinded to patient identity. All plasma samples from heterozygous carriers and NPC1 patients were collected following a period of fasting, whilst healthy control volunteers were unfasted.

Venous blood was collected into EDTA collection tubes and maintained at ambient temperature for a maximum duration of 72 hr. The blood was loaded onto an equivalent volume of Histopaque 1077 (Sigma-Aldrich), and centrifuged at 400 × *g* for 30 min. at room temperature. The plasma was then collected and frozen at −80 °C until ready for ^1^H NMR analysis (storage of samples prior to NMR analysis ranged from 9–1 yr.).

### Sample preparation

Samples were thawed and centrifuged for a period of 3.0 min. at 850 × g and 4 °C. A plasma volume of 200 μL was treated with 300 μL Mili-Q H_2_O and 55 μL D_2_O (the latter to provide a field frequency lock). Samples were then transferred to standard 5-mm diameter NMR tubes (Norell, USA) for analysis.

### ^1^H NMR analysis

NMR experiments on human plasma samples were performed on a Bruker AVIII 700-MHz spectrometer equipped with a ^1^H (^13^C/^15^N) TCI cryoprobe; sample temperature was stabilised at 310 K. ^1^H NMR spectra were acquired using a 1D NOESY presaturation scheme for attenuation of the water resonance with a 2 s presaturation. A spin-echo sequence (Carr-Purcell-Meiboom-Gill [CPMG]) with a τ interval of 400 μs, 80 loops, 32 data collections, an acquisition time of 1.5 s, a relaxation delay of 2 s, and a fixed receiver gain was employed to supress contributions from high-molecular-mass blood plasma components such as proteins which give rise to broad signals. The low-molecular-mass CPMG spectra were then used for multivariate analysis.

### NMR data preprocessing

Resulting free induction decays (FIDs) were zero-filled by a factor of 2 and multiplied by an exponential function corresponding to 0.30 Hz line broadening prior to Fourier transformation. All spectra were manually-phased and baseline corrected, and chemical shifts referenced to the lactate-CH_3_ function doublet located at δ = 1.33 ppm in Topspin 2.1 (Bruker, Germany). All spectra were visually examined for errors in baseline correction or referencing, and were then exported to ACD/Labs Spectrus Processor Academic Edition 12.01 (Advanced Chemistry Development, Inc.). Intelligent bucketing was applied to all spectra simultaneously with bucket widths of 0.04 ± 0.02 ppm. The intense H_2_O resonance (δ = 4.4–5.1 ppm) was excluded from all spectra acquired, together with any resonances arising from histopaque-derived agent contamination, i.e. those within the δ = 1.80–1.86, 2.18–2.31, 3.3–4.5, and 5.37–5.68 ppm regions. In addition, the δ = 1.15–1.17 ppm region was excluded in view of ethanol contamination, which probably arose from skin disinfection prior to sample collection. Regions which were shown to contain only noise across all spectra were also excluded in an effort to reduce the number of ^1^H NMR bucket variables for analysis and hence the discriminatory potential of models developed. Severely overlapping regions, such as the CH_2_-C**H**_**2**_-CH= and branched chain amino acid (isoleucine, leucine, and valine) regions, were manually bucketed to select regions with no overlap (isoleucine exclusively [0.913–0.936], leucine exclusively [0.938–0.960], and CH_2_-C**H**_**2**_-CH= lipid function exclusively [1.96–2.05]). It is not possible to measure the N-acetyl glycoprotein and proline resonances independently with this dataset, so these spectral bins are labelled N-acetyl glycoprotein/CH_2_-C**H**_**2**_-CH= [2.06–2.12] and proline/CH_2_-C**H**_**2**_-CH= [1.94–1.95] respectively, since contributions towards these from lipoprotein-associated triacylglycerols cannot be excluded. This resulted in a dataset containing 225 rows (75 untreated NPC1, 89 MGS treated NPC1, 31 HET, and 30 HC) and 38 potential predictor variable columns. Prior to statistical analysis, integral regions of each bucket were constant sum-normalised (CSN), cube root-transformed and Pareto-scaled. ^1^H NMR spectral resonances were assigned via a consideration of chemical shift values, coupling patterns and coupling constants, and confirmed by reference to literature values^37–41^ and the Human Metabolome Database (HMDB)^42–44^. These assignments were further confirmed via the acquisition of 2D total correlation spectroscopy (TOCSY) spectra on at least several samples in each disease classification. A full list of these assignments is provided in Table S1.

### Determination of lipoprotein and total cholesterol, and total triacylglycerol plasma levels using standard clinical chemistry techniques

Plasma samples were routinely analysed at the National Institute of Health using a standard clinical chemistry analyzer for a panel consisting of 32 standard tests including, among others, total cholesterol and total triacylglycerol levels.

### Univariate data analysis

All preprocessed ^1^H NMR data were imported into R (R foundation for statistical computing, Vienna, Austria). The spectral relative standard deviation was calculated as described previously^18^. In order to simultaneously compare all classifications, the post-hoc Tukey honest significant difference (HSD) test was performed following performance of analysis-of-variance (ANOVA). Corresponding *p* values obtained were then adjusted for multiple comparisons using the Bonferroni correction.

### Multivariate (MV) data analysis

Principal component analysis (PCA) was employed in order to obtain an overview of the degree of separation between/clustering of the disease classifications explored, and also to detect potential outliers. Investigation of the scores plots revealed no outliers. The random forests (RFs) technique was then employed for classification and variable selection using the randomForest R package^45^, with 1001 trees (ntree) and 7 predictors selected at each node (mtry) following tuning. Datasets were randomly split into training and test sets containing approximately two thirds and one third of them respectively. The training set was used to build the RFs model and obtain an out-of-the-bag (OOB) error value in order to assess the performance of the classification. The OOB error term is an estimate of the performance of the RFs model (i.e., how often the model classifies a sample incorrectly), and is computed using a test set (one third of the original dataset which is left out of the bootstrap sample used to construct the RFs model). The OOB error estimate ranges from 0 (a perfect model where 100% of the test set is correctly classified) to 1 (none of the test set was correctly classified). The test set was then used to determine the accuracy, specificity and sensitivity of this MV analysis strategy. This process was repeated 1000 times in order to prevent bias arising from the random sub-sampling of the training and test sets. The importance of each variable in the classification was determined by calculating the average mean decrease in accuracy (MDA) (using the OOB error observations) over all iterations. Discriminatory variables were then ranked in order of importance based on their mean MDA values, and further inspection of these values allowed identification of the number of variables required for classification purposes (variables with little or no change in MDA value were defined as redundant).

Canonical correlation analysis (CCorA) of PC scores vectors arising from separate PCA analyses of (1) ^1^H NMR lipoprotein-associated triacylglycerol resonance intensities (terminal-CH_3_ function resonance-normalised) and (2) clinical chemistry-determined total, LDL-and HDL-associated cholesterol concentrations (normalised to total TG level) was performed using XLSTAT2016 software, and a minimum filter factor of 90%. Preliminary PCA employed to acquire the scores vectors for CCorA was conducted both with a Varimax rotation (the latter with Kaiser normalisation and a maximum of 5 components), together with the application of Bartlett’s sphericity test.

## Electronic supplementary material

Supplementary Information
