# Supplementary material for: NMR analysis reveals significant differences in the plasma metabolic profiles of Niemann Pick C1 patients, heterozygous carriers, and healthy controls
Source: Sci Rep. 2017 Jul 24;7:6320. doi: 10.1038/s41598-017-06264-2 (PMC5524790; doi:10.1038/s41598-017-06264-2)
Supplement: Supplementary file 1 — Supplementary Information [file 41598_2017_6264_MOESM1_ESM.pdf]

NMR analysis reveals significant differences in the plasma metabolic profiles of Niemann Pick C1 patients, heterozygous carriers, and healthy controls.

Fay Probert[1,2], Victor Ruiz-Rodado[1], Danielle te Vruchte[2], Elena-Raluca Nicoli[2], Tim D.W. Claridge[4], Christopher A. Wassif[2,3], Nicole Farhat[3], Forbes D. Porter[3], Frances M. Platt[2] and Martin Grootveld[1*].

[1] Department of Pharmacology, De Montfort University, Leicester, UK.
[2] Department of Pharmacology, University of Oxford, Oxford, UK.
[3] Section of Molecular Dysmorphology, *Eunice Kennedy Shriver* National Institute of Child Health and Human Development, National Institute of Health, Department of Health and Human Services, Bethesda, MD, USA.
[4] Department of Chemistry, University of Oxford, Oxford, UK.

***Correspondence to [mgrootveld@dmu.ac.uk]**

Figure S1. Typical 700 MHz difference spectrum arising from the subtraction of a histopaque flow-through sample from that of its corresponding NPC1 plasma one. In view of the high level of variation in the level of contamination, subtraction of the histopaque signals did not permit observation and quantification additional resonances.

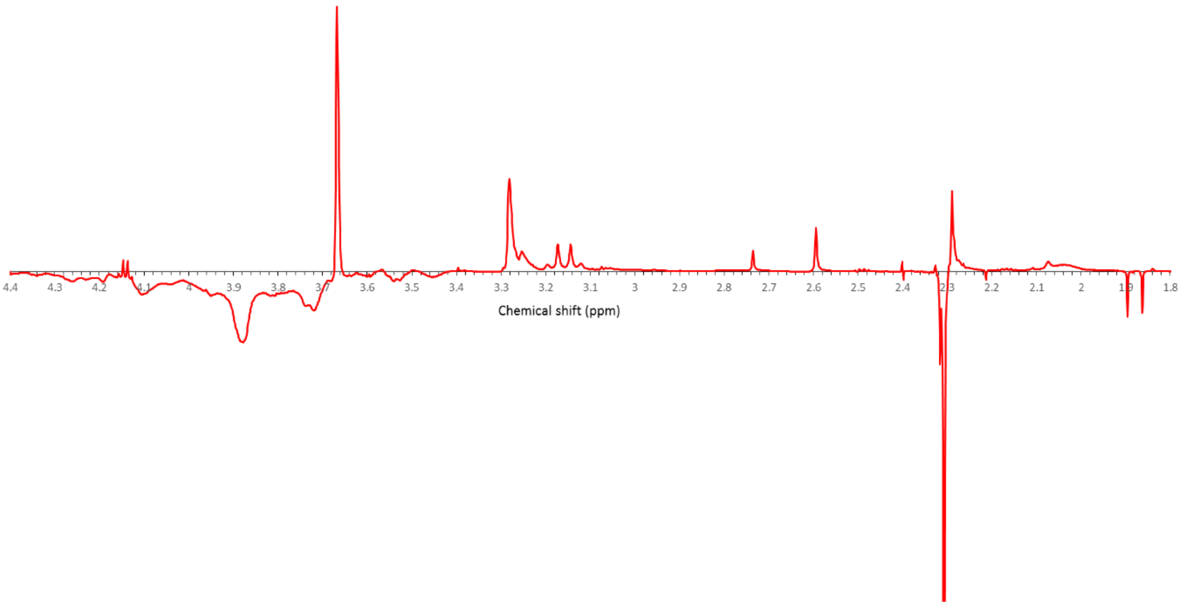

Figure S2. Box plots of (a) the spectral relative standard deviation of NPC1 (red), MGS (blue), HET (green) and HC (red) and (b) age distribution for female (F) and male (M) of each class.

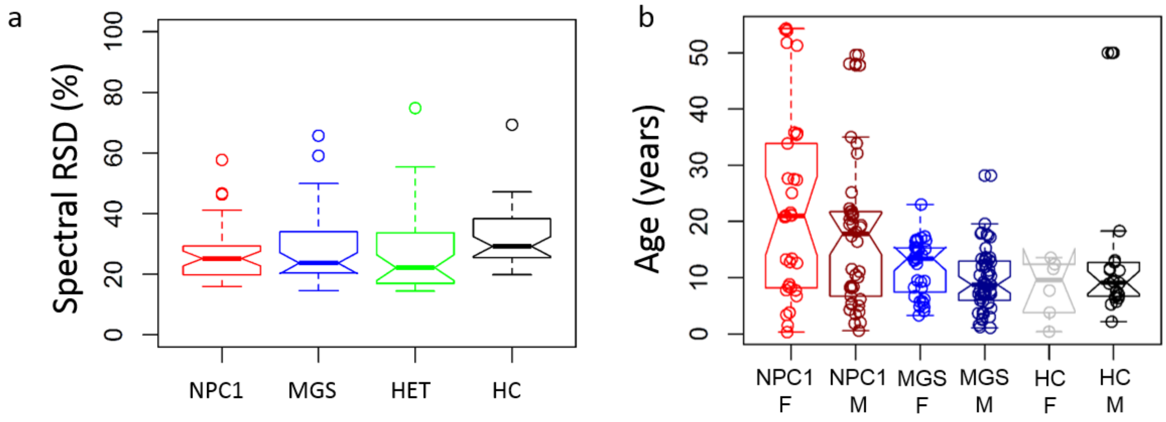

Figure S3. Average of all NPC1 patient plasma spectra form 0.8-2.1 ppm for (A) male (black) compared to female (red) and (B) less than 10 years old (red) and greater than or equal to 10 years old (black). Box plots show the median and range of the bucket integrals for the -CH_3 (VLDL) and -$(CH_2)_n$ (VLDL) regions of the spectra. There is no significant difference between gender or age in any region of the spectra. PCA scores plots for all NPC1 and HC samples showing no separation due to gender (C) or age (D).

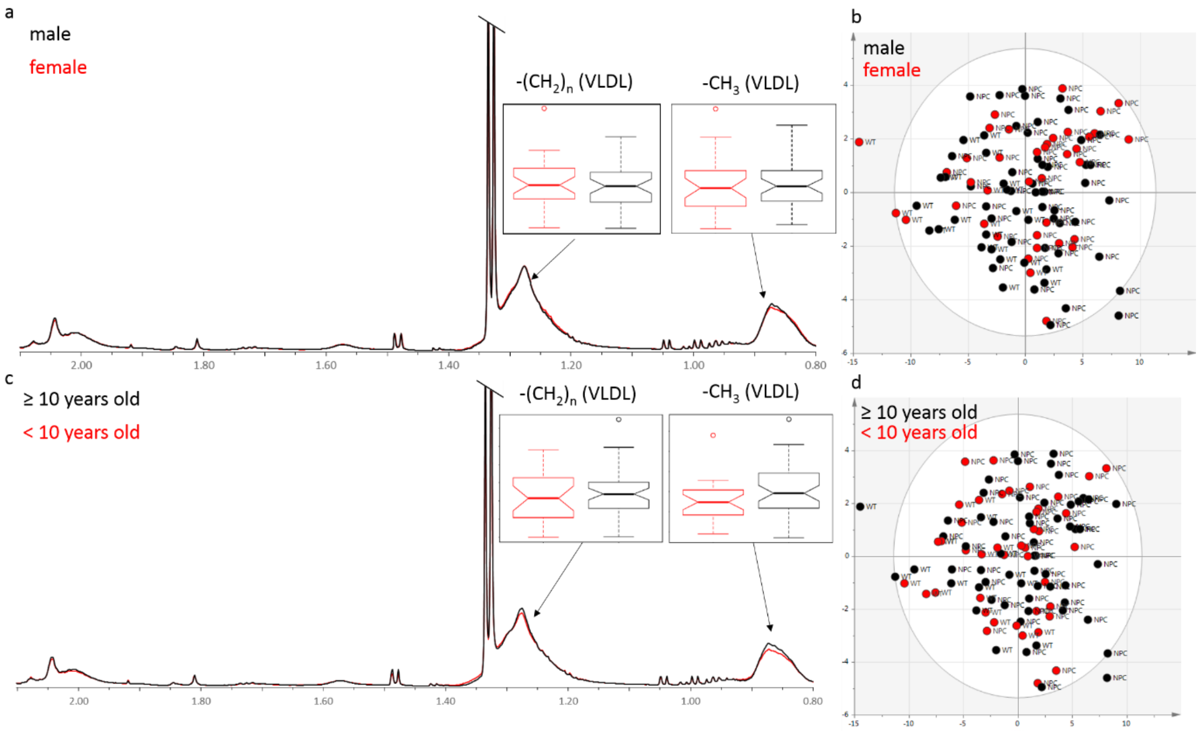

Figure S4. Plots of loadings on component 1 vs. component 2 for PCA models which showed distinctions between the a) NPC1 vs. HC, b) HET vs. HC, c) MGS vs. HC, and d) NPC1 vs. HET groups.

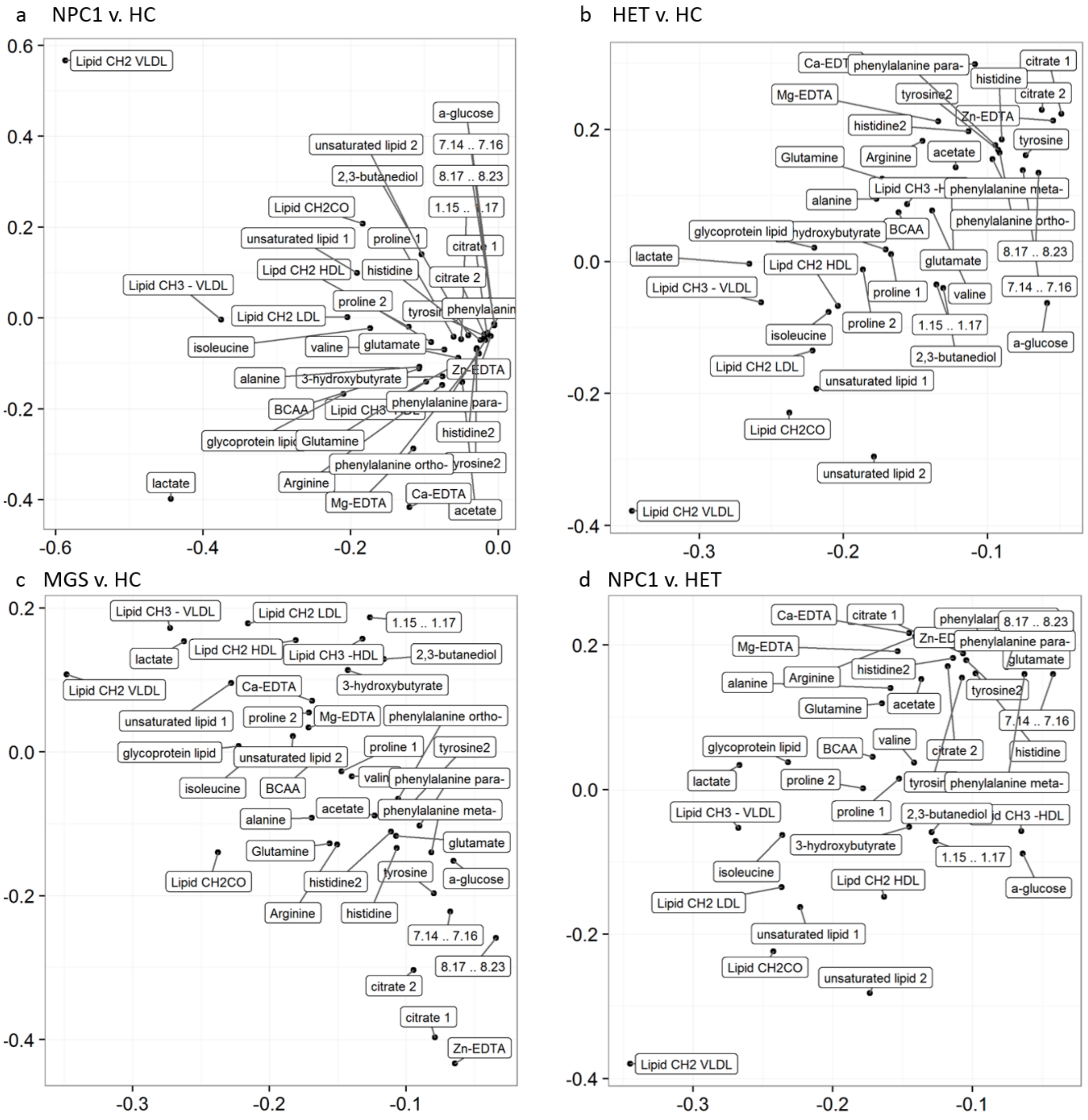

Figure S5. Box and whiskers plots illustrating the full range, interquartile range, and median of the Ca-EDTA spectral intensities of the NPC1 (red), MGS (blue), HET (green) and HC (black) plasma samples (Bonferroni corrected *p*-values <0.05, 0.01, 0.001 are represented by *, **, and *** respectively).

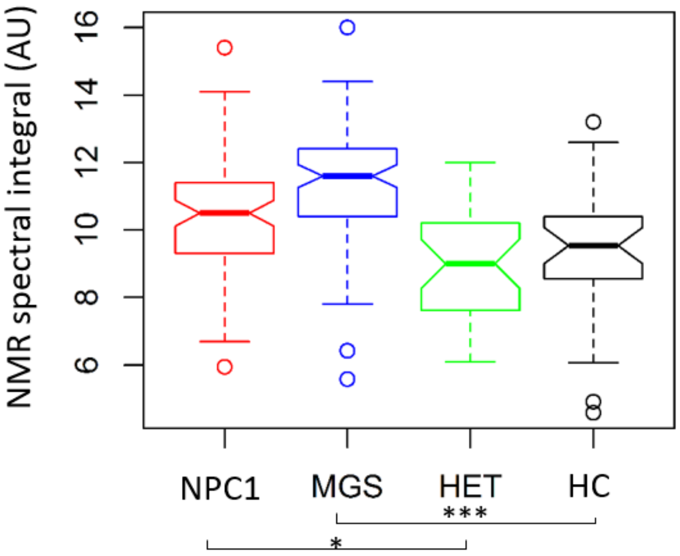

Table S1. Univariate comparisons of variables, displaying results from the performance of Tukey's *HSD* test for every metabolite tested in this context. Statistical significance is indicated by asterisks (Bonferroni corrected *p*-values <0.05, 0.01, 0.001 are represented by *, **, and *** respectively). Fold-changes are given with respect to the HC samples, or with respect to the Het or MGS samples in the case of the NPC1 v. Het and NPC1 v. MGS comparisons respectively.

**Lipids**	HC v. NPC1	HC v. Het	HC v. MGS	NPC1 v. Het	NPC1 v. MGS
$-CH_3$ (HDL)					
$-CH_3$ (VLDL)	1.26***	0.96***	1.13***		
$-(CH_2-)_n$ (HDL)	1.40*	0.84***	1.06***		
$-(CH_2-)_n$ (LDL)	1.11***	0.62***	1.38***		
$-(CH_2-)_n$ (VLDL)	1.1***	0.54***	1.41***		
$-C\mathbf{H_2}CH_2CO$	0.86***	0.58**	1.6***		
$-CH_2-C\mathbf{H_2}-CH=$ (exclusively unsaturated lipid function)	1.07***		1.31***		
Unsaturated lipid >CH=CH< (bin 1)	0.91***	1.2***	1.34***		
Unsaturated lipid >CH=CH< (bin 2)	1***	1.48***	1.17***		
**Amino acids**					
isoleucine	1.18***		1.14***		
leucine					
valine	1.25**		1.23***		
alanine	1.17***	1.05*	1.17***		
arginine	1.41***		1.02***		
Proline/$-CH_2-C\mathbf{H_2}-CH=$ (bin 1)	1.22***	0.62***	1.28***		
proline/$-CH_2-C\mathbf{H_2}-CH=$ (bin 2)	1.6***	0.33***	1.06***		
glutamine	1.25***		1.04***		
glutamate					
tyrosine (bin 1)					
histidine (bin 1)	1.26**		1.17***		
tyrosine (bin 2)					
phenylalanine (ortho-)	1.63*		1.34*		
phenylalanine (para-)					
phenylalanine (meta-)			1.81*		
histidine (bin 2)	1.04**		1.49**		
Overlapping N-acetyl glycoprotein/$CH_2-C\mathbf{H_2}-CH=$					
**Metal ions**					
Ca^{2+}-EDTA			0.64***	1.88*	
Mg^{2+}-EDTA			1.28***		
Zn^{2+}-EDTA					

**Other**					
2,3-butanediol					
[1.15 .. 1.17]	1.52***		1.19***		
3-hydroxybutyrate			1.38*		
acetate					
citrate 1					
citrate 2					
a-glucose					
[7.14 .. 7.16]					
[8.17 .. 8.23]					
